# Macroecological diversification of ants is linked to angiosperm evolution

**DOI:** 10.1093/evlett/qrad008

**Published:** 2023-03-31

**Authors:** Matthew P Nelsen, Corrie S Moreau, C Kevin Boyce, Richard H Ree

**Affiliations:** The Field Museum, Negaunee Integrative Research Center, Chicago, IL, United States; The Field Museum, Negaunee Integrative Research Center, Chicago, IL, United States; Departments of Entomology and Ecology & Evolutionary Biology, Cornell University, Ithaca, NY, United States; Department of Geological Sciences, Stanford University, Stanford, CA, United States; The Field Museum, Negaunee Integrative Research Center, Chicago, IL, United States

**Keywords:** plant–insect interaction, macroevolution, angiosperms, ants

## Abstract

Ants are abundant, diverse, and occupy nearly all habitats and regions of the world. Previous work has demonstrated that ant diversification coincided with the rise of the angiosperms, and that several plant traits evolved as ants began to expand their nesting and foraging habits. In this study, we investigate whether associations with plants enabled niche expansion and are linked to climatic niche evolution in ants. Our analysis of over 1,400 ant species reveals that ancestral expansion from forest floors into the canopy and out into non-forested habitats closely followed evolutionary innovations in angiosperms. Several Paleogene-Neogene ant lineages independently diversified in non-forested habitats on multiple continents, tracking the evolution and expansion of elaiosome-bearing and arid-adapted angiosperms. The evolution of arboreal nesting tracked shifts in angiosperm physiology associated with the onset of everwet tropical rainforests, and climatic optima and rates of climatic niche evolution were linked to nesting location, with arboreally nesting groups having warmer and less seasonal climatic optima, and lower rates of climatic niche evolution. Our work further underscores the varied paths by which niche diversification occurred in ants, and how angiosperms influenced the ecological and evolutionary trajectories of interacting lineages.

## Introduction

Shifts in vegetation may be driven by climate change and by evolutionary innovations. Such turnover in vegetation may drive feedbacks that modify climate or facilitate the expansion of other lineages dependent on these plants via the climates the plants help engender (Boyce et al., 2010; Boyce & Lee, 2010, 2016; Moreau et al., 2006; Schuettpelz & Pryer, 2009). Ants are exemplary in this regard. Modern ants include over 14,000 species and are some of the most abundant insects on earth (Wilson, 1988). Their diversity is concentrated in forests, where modern ants originated (Dunn et al., 2007; Economo et al., 2018; Jenkins et al., 2011; Moreau & Bell, 2013; Perrichot et al., 2008; Wilson & Hölldobler, 2005), but some modern species occupy non-forested (open-canopy) habitats, such as savannas, grasslands and deserts, where they play important roles in nutrient cycling via soil bioturbation and predation, structuring plant communities through seed consumption and dispersal, and the chemical and physical modification of soil (Beattie, 1989; Beattie & Hughes, 2002; Folgarait, 1998; Hölldobler & Wilson, 1990). When did ants begin to exploit and diversify in non-forested biomes?

While non-forested biomes pre-date the Early Cretaceous origin of ants (Beerling & Woodward, 2001; Rees et al., 1999), their range, composition, and structure has changed through time. For instance, angiosperm-dominated grasslands and deserts expanded through the late Paleogene-Neogene (Leopold et al., 1992; Singh, 1988; Strömberg, 2011; Willis & McElwain, 2014). Plant diversity in open habitats may have been low and largely restricted to non-angiosperms until the Paleogene-Neogene, when several angiosperm lineages evolved physiological adaptations to hot, arid habitats—such as C4/CAM photosynthesis—or invaded open habitats (Arakaki et al., 2011; Bouchenak-Khelladi et al., 2010; Christin et al., 2014; Edwards et al., 2010; Horn et al., 2014; Leopold et al., 1992; McKain et al., 2016; Onstein et al., 2016; Singh, 1988; Strömberg, 2011; Ziegler et al., 2003).

Elaiosomes and extrafloral nectaries (EFNs) can provide important food sources for ants (Aranda-Rickert et al., 2014; Beattie & Hughes, 2002; Hölldobler & Wilson, 1990; Johnson, 2001; Lengyel et al., 2010, 2009; Pemberton, 1988; Rico-Gray & Oliveira, 2007; Weber & Agrawal, 2014; Weber & Keeler, 2013), and the evolution specifically of elaiosomes is hypothesized to have facilitated the expansion of ants into open-canopy biomes such as deserts and dry grasslands (Wilson & Hölldobler, 2005), with plants in turn acquiring protection and dispersal services from the ants to nutrient-rich locations while reducing parent-offspring conflict (Lengyel et al., 2009, 2010; Turner & Frederickson, 2013). Indeed, subsequent work has demonstrated the increased evolutionary potential of numerous angiosperm lineages to form elaiosomes and EFNs during the Paleogene-Neogene (Nelsen et al., 2018), including known open-habitat angiosperms commonly dispersed by ants, such as Cactaceae (Arakaki et al., 2011; Bregman, 1988), Proteaceae—occupying Cretaceous arid habitats, but only later evolving elaiosomes during the Paleogene-Neogene (Lamont & He, 2012)—and certain *Euphorbia* species (Bruyns et al., 2011; Horn et al., 2012, 2014). Similarly, other insect lineages that occupy these habitats and rely on grasses also began evolving in the early Neogene (Kergoat et al., 2018), and could conceivably have served as food sources to predatory ants.

The confluence of the expanding open-canopy angiosperm-dominated ecosystems, the evolution of physiological adaptations in angiosperms, and the evolution of novel angiosperm-derived food sources for ants thus highlights the Paleogene-Neogene as an important period for the potential colonization of open-canopy habitats by ants. A Paleogene expansion of ants into non-forested habitats—tracking the angiosperm invasion of these habitats—has been hypothesized (Wilson & Hölldobler, 2005), but has not been rigorously tested. Advances in our understanding of ant phylogeny and evolution in the interim motivate a more detailed evaluation of the timing and potential drivers of ant diversification in non-forested biomes.

In addition to the outward expansion from forested habitats, ants also moved upward from the ground and evolved to nest arboreally. Nesting space is one of the most important resources for ants; it is where the queen is sheltered, the brood is reared, and food is stored and exchanged (Blüthgen & Feldhaar, 2010). While arboreally nesting ants occur in a wide range of climates, they are most abundant and diverse in the tropics where they comprise a greater proportion of the local ant biota (Blüthgen & Feldhaar, 2010; Dejean et al., 2007; Floren et al., 2002, 2014). Their reduced importance or complete absence from temperate and boreal habitats may be because ground-nesting provides a more stable, buffered environment in which humidity levels and temperature remain less variable throughout the year, and are far more favorable in seasonal habitats (Blüthgen & Feldhaar, 2010). In addition to greater thermal variability, water stress levels in a forest canopy may be comparable to those experienced in deserts (Hood & Tschinkel, 1990). Such extremes may be limiting to ants in more temperate regions (Floren et al., 2014; Hölldobler & Wilson, 1990; Majer, 1990; Seifert, 2008).

Like most modern ants, the earliest ants were ground-nesting, with the evolution of arboreal nesting proceeding in Late Cretaceous-Paleogene (Lucky et al., 2013; Nelsen et al., 2018). One expectation may be that the ecological or physiological constraints associated with ground versus arboreal nesting may be reflected in the trajectories of climatic niche evolution in ants. Much like the tropical conservatism hypothesis (Wiens & Donoghue, 2004), the occupation of warmer and wetter climates by arboreal nesters may be the result of their climatic occupancies evolving under a selective regime characterized by low rates of evolutionary drift or strong stabilizing selection. Alternatively, the collectively broad range of climates occupied by ground-nesting ants may instead be a consequence of their older age, rather than contrasting evolutionary dynamics. Here, we explicitly compare these two competing hypotheses by modeling the evolution of climatic occupancy and determine whether arboreal- and ground-nesting lineages occupy distinct adaptive optima, and whether nesting location is linked to varying rates of, and constraints on, climatic niche evolution.

Our overarching goal is to study the ecological diversification of ants in the context of angiosperm and biome evolution. More specifically, we seek to understand: (1) whether ants began to rely extensively on, and to diversify in, non-forested habitats during the Paleogene-Neogene; and (2) whether ant nesting location modulates the evolution of climatic occupation. Our results strengthen our understanding of the diverse and complex ways by which ants evolved to occupy disparate habitats, and how angiosperms likely shaped their evolution.

## Materials and methods

### Occurrence data

To characterize the climatic niche of extant ants and model its evolution, we first obtained occurrence data with geographic coordinates that enabled us to extract climatic and biome data for individual occurrences. Occurrence records for ant species in the 2017 Bolton checklist of valid species (AntWiki, 2017) were obtained by querying AntWeb (AntWeb, 2017) using the AntWeb (Ram, 2014) package in R (R Core Team, 2014). This approach provided a rapid and simple means by which to obtain detailed locality information. Records with unique coordinates were retained for each species, reduced to include species present (1435) in a previous species-level phylogeny of ants (Nelsen et al., 2018). We then used a modified version of functions in the rangeBuilder (Davis Rabosky, 2017) package to ensure coordinates were from or near the country in which they were reported. This function also reverses and flips coordinates if they are not over the country expected, and also ensures coordinates are over land. Records not passing these requirements were excluded.

Geographic coordinates were then used to extract information for 36 environmental (19 bioclimatic variables [bioclim] (Hijmans et al., 2005), net primary productivity, potential evapotranspiration [PET], elevation) and topsoil (percent gravel, sand, silt, clay, bulk density, organic Carbon, pH, CEC, BS, TEB, CaCO_3_, CaSO_4_, ESP, ECE) variables from various sources (Supplementary Table S1). Temperature-related bioclim variables were converted to degrees Celsius by dividing by 10. Occurrences were only retained if data for all variables could be acquired. For each taxon, median values for individual bioclimatic variables were then calculated for use in downstream analyses. Our dataset included 1,435 species with 58,424 occurrence records (min = 1 [193 taxa]; mean = 40.7; SD = 95.3; max = 1,134 [2 taxa]). Vegetative biome and realm type (Olson et al., 2001) was extracted (Supplementary Table S1) for each unique location, and the proportion of unique records occurring in each category were tabulated for each species.

### Phylogeny

A previously published phylogeny (Nelsen et al., 2018), representing the most complete sequence-based, species-level molecular phylogeny of ants, was used for subsequent analyses. This phylogeny was previously timescaled (Nelsen et al., 2018) using penalized likelihood (Smith & O’Meara, 2012) together with 51 fossil calibration points (minimum ages) (Barden, 2017), a 185 Ma fixed age constraint for the root (Brady et al., 2006), and maximum ages for all calibrated nodes set to 185 Ma. Age estimates of major clades in this phylogeny broadly agree with estimates derived from studies relying on fewer taxa and/or calibration points (Blanchard & Moreau, 2017; Borowiec et al., 2020; Brady et al., 2006; Moreau et al., 2006; Moreau & Bell, 2013; Schmidt, 2013). Because of uncertainty in age estimates across and within studies, we discuss our findings in coarse temporal time bins.

### Diversification in non-forested biomes—ancestral state reconstruction

We extracted vegetative biome type from coordinate data and reduced individual biomes to a binary canopy type in which biome canopy types were considered open (non-forested) or closed (forested) (Supplementary Table S2). Biome types that typically included a more or less continuous canopy of trees were treated as having a closed canopy, and are also referred to as forested, while those lacking this were regarded as having an open canopy and regarded as non-forested habitats. The proportion of unique occurrences of each taxon in open or closed habitats was calculated to determine the occurrence frequency in each habitat type. Taxa were first scored for two binary characters: the occupation of forested habitats with a closed canopy, and occupation of non-forested habitats with an open canopy. If over 1/3 of unique records were found in biomes with closed canopies, they were scored positively as occupying closed canopy habitats, and if over 1/3 of the unique records derived from open-canopy biomes, they were coded as occupying open canopy habitats. From these two binary characters, a new character for canopy type was generated in which taxa coded as overwhelmingly occupying closed canopy habitats (state 0, with character state combination: closed = 1, open = 0), occupying both habitats (state 0&1, with character state combination: closed = 1, open = 1), or overwhelmingly occupying open canopy habitats (state 1, with character state combination: closed = 0, open = 1). We then modeled the evolution of habitat type (open canopy, open & closed canopy, closed canopy) in corHMM (Beaulieu & O’Meara, 2017) using the rayDISC function. Our model prohibited direct transitions between specialized closed canopy (0) and open canopy (2) habitats, and instead required transition through the more generalized state (1) in which taxa occupied both closed and open canopy habitats. Transition rates were estimated under “all rates different” (ARD) model, and internal node state probabilities were subsequently inferred using marginal reconstruction, while root state probabilities were set using the “maddfitz” method (FitzJohn et al., 2009). As Mediterranean forest, woodland and scrub communities are sometimes considered to be grassland communities (Gibson, 2009), we performed an alternate set of analyses in which these were treated as open habitat.

### Modeling the evolution of climatic occupancy

To assess how climatic niche occupancy evolved in ants, we fit several models of evolution to median estimates of six climatic variables: annual mean temperature (BIO1), temperature seasonality (BIO4), minimum temperature of the coldest month (BIO6), temperature annual range (BIO7), mean temperature of the coldest quarter (BIO11), and annual precipitation (BIO12) for 1417 species that had environmental data and nesting location (see below). These variables were selected as they represent different measures of temperature, seasonality, and water limitation, which play strong roles in structuring ant distributions (Pie, 2016).

We fit Brownian motion (BM) and Ornstein-Uhlenbeck (OU) models, and then tested whether the rate and model of evolution of climatic niche occupancy was linked to nesting position. We used OUwie (Beaulieu & Donoghue, 2013) to fit trait-independent BM and OU models, as well as five trait-dependent BM and OU models which varied by having trait-dependent estimates of evolutionary rate or optima (θ) (BMS, OUM). Additionally, OU models with a trait-dependent optima (θ) were fit with single and/or state-dependent rates of adaptation, α (A), and rates of evolution, σ^2^ (V) (OUMV, OUMA, OUMVA). Here, we emphasize that our definition of optima is based on known occurrences and coarse-grained estimates of temperature, and is regarded as an evolutionary optimum, rather than fine-scaled estimates and experimentally validated tolerances and physiological optima (Kaspari et al., 2015). If the younger age of arboreally nesting ants was the sole reason for their limitation to habitats with narrow annual climatic variability, then the favored models are expected to be those in which rates of evolution, climatic optima and selection strength are shared among ground- and arboreal-nesting lineages. In contrast, support for a state-dependent model would suggest that climatic niche evolution may be underlain by different evolutionary processes and constraints.

Nesting position was derived from (Blanchard & Moreau, 2017) and modifications of (Nelsen et al., 2018). The tree and ancestral state estimates for this character were derived from (Nelsen et al., 2018) and recoded from a multi-state character representing diet, foraging and nesting location, to a binary nesting character (strictly ground nesting vs. arboreal or arboreal and ground nesting). Taxa that were ambiguous (scored as “?” [Blanchard & Moreau, 2017; Nelsen et al., 2018]) or lacking environmental and soil data were removed from the tree, resulting in a dataset of 1,417 species. Following the associated documentation, the starting state (θ_0_) was estimated in all analyses except the BMS model. As fitting some models can be especially problematic, we performed diagnostic analyses, and checked that all eigenvalues in the Hessian matrix were greater than 0. AICc values and weights were then compared to identify the best-fit model. In the case of OU models, we calculated the phylogenetic half-life (ln(2)/α), which represents the time required for trait value to move halfway from the ancestral value to the optimum (Hansen, 1997), and the stationary variance (σ^2^/(2α)), which represents the equilibrium variance under a stationary optimum (θ). For each trait, we then performed parametric bootstrapping by simulating 50 datasets under the best-fit model and the estimated parameter values, fit this model to the data, and calculated 95% confidence intervals for each parameter.

## Results

### Diversification in non-forested habitats

**Figure 1.**
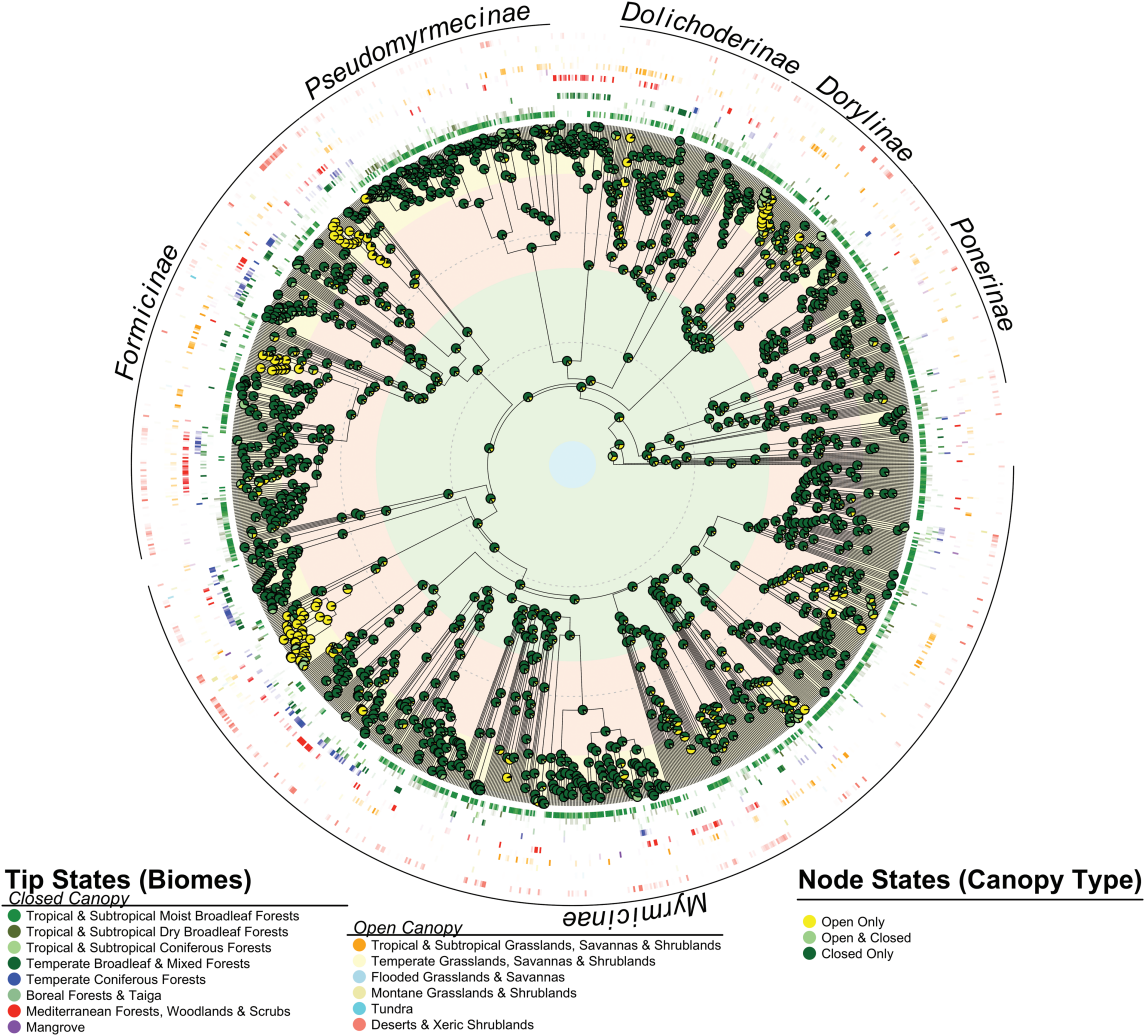
Time-scaled phylogeny of 1,435 ant species illustrating the evolution of ants in open- and closed-canopy habitats (non-forested and forested, respectively). Pie charts overlaying nodes indicate the proportional probability of occupying closed canopy, open canopy or mixed habitats. Rings around the tips of the phylogeny are colored by modern biome type and opaqueness reflects the proportion of specimens for individual species recovered from each biome. Shaded bands under the phylogeny correspond to geological periods, with dashed lines occurring in 50-Ma intervals.

Ancestral state reconstruction of the root node yielded ambiguous results for whether the ancestor of extant ants occupied forested or non-forested habitats (Figure 1, Supplementary Figure S1). Our work suggests that most early ant lineages likely occupied forested habitats until around the middle-late Paleogene-early Neogene, when several clades independently evolved specialized preferences for non-forested habitats and continued to diversify in them through the Neogene. These transitions occurred in lineages whose extant members may be abundant in deserts and xeric shrublands (Formicinae, Myrmicinae), and tropical and subtropical grasslands, savannas and shrublands (Dorylinae, Formicinae, Myrmicinae). Results changed slightly if Mediterranean forests, woodlands and scrubs were treated as forested despite their mixed to open canopies, and suggested that fewer lineages may have evolved a full reliance on open habitats during the late Paleogene-early Neogene (Supplementary Figure S2). Together, these findings demonstrate that the wholesale reliance on—and diversification in—non-forested habitats occurred relatively recently and independently in several clades.

### Relationship between nesting location and the evolution of climatic occupancy

**Figure 2.**
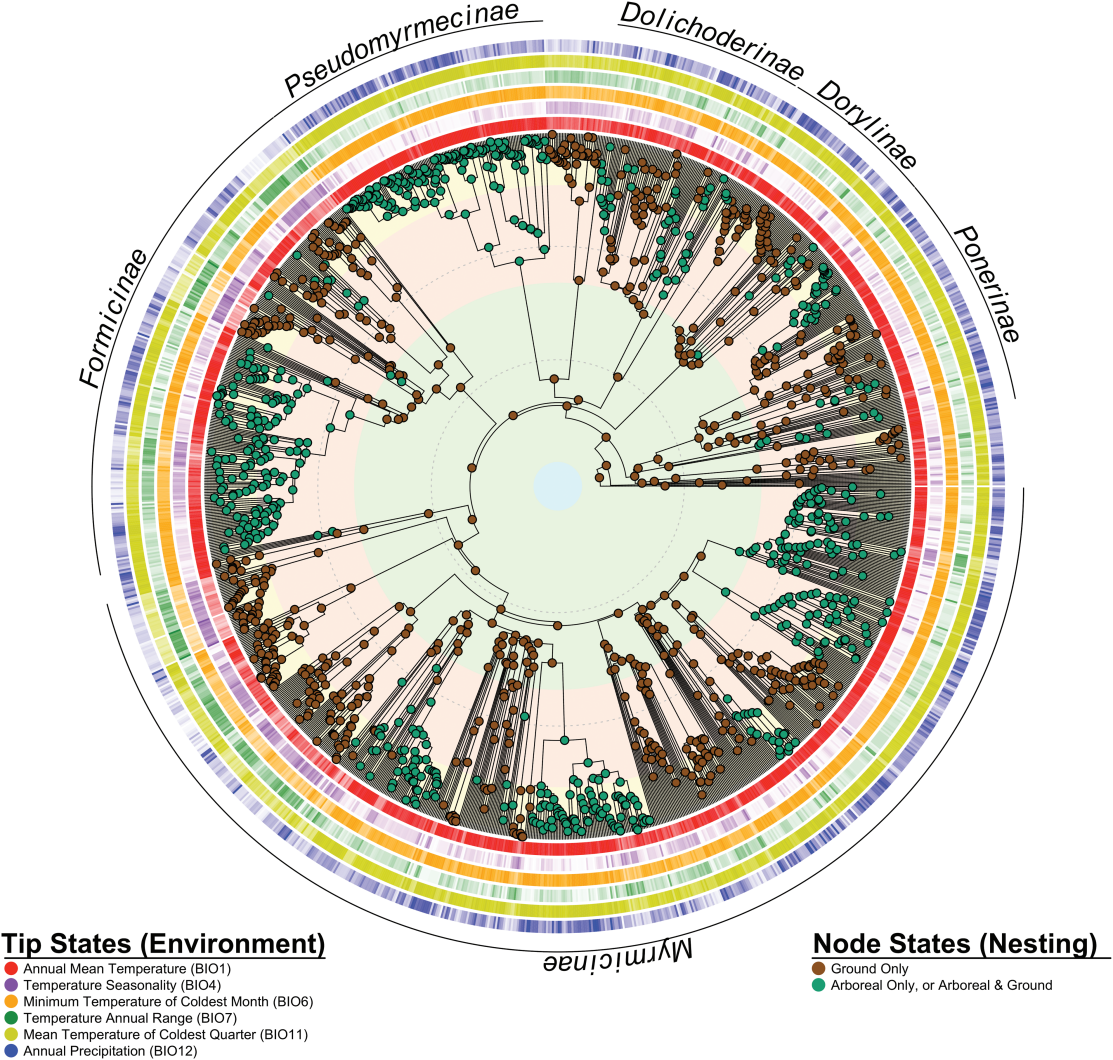
Time-scaled phylogeny of 1,417 ant species illustrating the evolution of nesting location and the climatic occupancy of individual species. Nodes are colored by the most-likely nesting location (derived from (Nelsen et al., 2018)). Rings around the tips of the phylogeny are colored by environmental variable, and opaqueness is proportional to the median value of each species with white corresponding to the minimum value (BIO1 = −2.1; BIO4 = 15; BIO6 = −28.4; BIO7 = 7.4; BIO11 = −20.6; BIO12 = 64), and color in legend for each variable to the maximum value (BIO1 = 28.5; BIO4 = 1,405.3; BIO6 = 22.7; BIO7 = 51.3; BIO11 = 27.4; BIO12 = 4,506). Shaded bands under the phylogeny correspond to geological periods, with dashed lines occurring in 50-Ma intervals.

For all six climatic variables (Figure 2; Supplementary Table S3), the OUMVA (Ornstein-Uhlenbeck [OU] with trait dependent optima [M, or θ], rate of adaptation [A, or α], and rate of evolution [V, or σ^2^]) model was recovered as the best fit (Supplementary Table S3). The OUMA model fit to BIO12 was excluded as it failed the diagnostic tests. We then simulated data under the OUMVA model for all variables using the parameter estimates obtained for the observed data and fit the OUMVA model to the simulated data. All datasets were fit without analytical issues, and 95% confidence intervals were calculated. Observed values always fell within the 95% confidence intervals with the exception of the stationary variance for the ground-nesting state in BIO4 and BIO7 (Supplementary Table S4). Arboreally nesting groups had thermal optima (θ) that were significantly warmer (BIO1, BIO6, BIO11) and less seasonal (BIO4, BIO7) than ground nesting lineages, and evolved at significantly lower rates (σ^2^) than those of ground nesting lineages. In addition, the optimum for the precipitation variable investigated (BIO12) was significantly wetter for arboreal nesting groups. Stationary variance for arboreal-nesting groups was significantly lower for all variables except BIO12, for which their distributions overlapped. Despite OUMVA always being the best-fit, selection strength (α) and the phylogenetic half-lives (ln(2)/α) did not vary significantly among nesting groups. It was not immediately clear why the more complex model (OUMVA) was favored over OUMV, when parametric bootstrapping did not reveal a significant difference among state-dependent selection strengths.

## Discussion

### Diversification in non-forested habitats

While non-forested habitats, such as deserts, have always been available for ants to occupy, our analyses suggest many of the earliest ant lineages were likely restricted to forested habitats during the Cretaceous (Figure 1), as previously anticipated (Wilson & Hölldobler, 2005). Climate and vegetation reconstructions for this time period suggest most forests would have been confined to the cool and wet mid-high latitudes, while lower latitudes were covered with hot and dry tropical savanna woodland and desert, along with a narrow wet belt extending across the equator that could have harbored deciduous forests (Saward, 1992; Upchurch et al., 1999; Ziegler et al., 2003).

By contrast, our analyses demonstrate that ants did not extensively diversify in non-forested habitats until the middle-late Paleogene-early Neogene (Figure 1). This was likely associated with the evolution, sustained diversification, and spread of dry-adapted or seasonal, open-habitat angiosperms, that could have provided food sources to ants while simultaneously increasing diversity and habitat complexity. The Paleogene-Neogene diversification of open-habitat grasses and grass-dominated ecosystems in several distinct geographic regions account for most of the non-forested biomes considered here (Gibson, 2009; Olson et al., 2001). North American open habitat grasses are known from the late Paleogene together with grass-dominated habitats from the Paleogene-Neogene boundary (Strömberg, 2011); this is consistent with the presence of Neogene fossilized nests attributed to seed-harvesting *Pogonomyrmex* ants (Smith et al., 2011), and to our inferred late Paleogene occurrence of open habitat lineages (such as *Pogonomyrmex* [Myrmicinae], *Myrmecocystus* [Formicinae]) with a North American distribution (Guénard et al., 2017; Janicki et al., 2016) (Figure 1, Supplementary Figure S1). Similarly, African open-habitat grasses and grass-dominated ecosystems are suggested from the late Paleogene (Bouchenak-Khelladi et al., 2010) and early-mid Neogene (Strömberg, 2011), respectively, consistent with the early Neogene evolution and diversification of an open habitat Dorylinae (*Dorylus*) lineage with an African distribution and center of diversity (Janicki et al., 2016; Guénard et al., 2017) (Figure 1, Supplementary Figure S1). Open-habitat grasses were also present in South America by the late Paleogene, with grasslands evolving in the mid-Neogene (Strömberg, 2011; Strömberg et al., 2013); this is again loosely consistent with the Paleogene-Neogene origination and subsequent diversification of an open habitat Myrmicinae lineage (several *Cephalotes* spp.) that occurs throughout South America (Guénard et al., 2017; Janicki et al., 2016) (Figure 1, Supplementary Figure S1). Finally, open-habitat grasses from Australia may have evolved by the early-mid Neogene, with grass-dominated ecosystems arising during the late Neogene (Strömberg, 2011); this is slightly later than our Paleogene-Neogene inferred presence of an open-habitat Formicinae lineage (several *Polyrhachis* spp.) in Australia (Guénard et al., 2017; Janicki et al., 2016), but post-dates the evolution of non-graminoid, open-habitat *Banksia* species (Onstein et al., 2016). Refined molecular clock analyses of *Polyrhachis* may yield dates more in line with grass fossil data or new fossil data may yield a slightly older age for Australian open-habitat grasses. Future work including more taxa, outgroups, fossil data, and ancestral ranges may resolve ambiguities across the phylogeny and in the root state.

### Angiosperms, climate, and the evolution of arboreal nesting

We demonstrate that while early ants likely occupied forested habitats, they constructed their nests in the ground instead of the surrounding trees. The subsequent Late Cretaceous-early Paleogene shifts to arboreal nesting increased the stratification of communities, while likely reducing interspecific competition and facilitating species coexistence in greater numbers. These convergent transitions to arboreal nesting were geographically widespread, as indicated by independent biogeographic reconstructions of lineages such as *Cephalotes* and *Tetramorium* (Ward et al., 2015).

Our analyses also demonstrate that the evolutionary trajectories of climatic occupancy are linked to nesting location. For instance, climatic optima of arboreally nesting lineages were wetter, warmer and experienced reduced thermal variability than those of ground-nesting lineages. These transitions to arboreal nesting broadly coincided with major physiological changes in angiosperm leaf hydraulics that increased leaf gas exchange capacity and, thus, water loss—ultimately increasing the abundance and reliability of precipitation while reducing seasonality in the tropics (Boyce & Lee, 2010; Boyce & Lee, 2016; Boyce et al., 2010; Feild et al., 2011). Thus, the habitats favored by arboreally nesting ants—wet, aseasonal tropical rainforests—likely expanded during or after the Late Cretaceous-early Paleogene, when angiosperms evolved leaf hydraulics comparable with those in modern tropical rainforest taxa (Feild et al., 2011). A similar and contemporaneous pattern played out with the repeated evolution of epiphytic plants (such as ferns, liverworts, orchids, and bromeliads) over the same timeframe (Boyce et al., 2010; Feldberg et al., 2014; Givnish et al., 2014, 2015; Schuettpelz & Pryer, 2009). In part, the ants and other canopy-based organisms may be separately responding to the same angiosperm-driven changes to moisture regimes that were more permissive of canopy occupation. However, epiphytic plants also frequently provide ants with specialized structures for nesting and extrafloral nectar for food (Blüthgen et al., 2000; Lüttge, 2008); thus their evolution continued to benefit arboreal ants. Shifts to arboreal nesting also broadly coincided with, or slightly preceded, the Cenozoic evolution of arboreality in desiccation-intolerant lineages including: frogs (Feng et al., 2017), snakes (Harrington et al., 2018; Zheng & Wiens, 2016) and salamanders (Baken & Adams, 2019). As ants may comprise a substantial proportion of arthropod individuals and biomass (sometimes over 70%) in the canopies of tropical forests (Davidson & Patrell-Kim, 1996; Erwin, 1983; Tobin, 1995), their evolution likely further benefited the evolution of insectivores, such as the arboreal and mymecophagous Eocene ancestor of modern anteaters (Casali et al., 2020; Gaudin & Branham, 1998; Gaudin & Croft, 2015; Gibb et al., 2016; Toledo et al., 2015). Together, this temporally restricted development provides further support for the climatic influence of angiosperm physiology and the development of the canopy as a habitable environment capable of sustaining complex arboreal communities and food webs.

## Conclusions

This study furthers our understanding of how ants convergently evolved to occupy similar climatic regimes and habitat types in geographically disparate regions, that were likely facilitated by physiological and anatomical innovations in angiosperms. Ant diversification in non-forested habitats broadly coincided with the evolution of photosynthetic pathways enabling angiosperms to invade these habitats, and the evolution of plant-derived food sources for ants. Arboreally nesting lineages occupy warmer, wetter and less seasonal climates than ground-nesting lineages, and diversified as angiosperms facilitated the evolution of these climates and complex arboreal communities. Together, this work further illustrates the complex and diverse means by which angiosperms likely enabled the ecological diversification of ants (Moreau et al., 2006; Wilson & Hölldobler, 2005).

## Supplementary Material

qrad008_suppl_Supplementary_Material

qrad008_suppl_Supplementary_Table_S4

## Data Availability

No new data were generated in this study. Data and code are available on GitHub https://github.com/mpnelsen/Nelsen_et_al_2023_Evolution_Letters_Ants).
