# Supplementary material for: Macroecological diversification of ants is linked to angiosperm evolution
Source: Evol Lett. 2023 Mar 31;7(2):79–87. doi: 10.1093/evlett/qrad008 (PMC10078972; doi:10.1093/evlett/qrad008)
Supplement: qrad008_suppl_Supplementary_Material [file qrad008_suppl_supplementary_material.pdf]

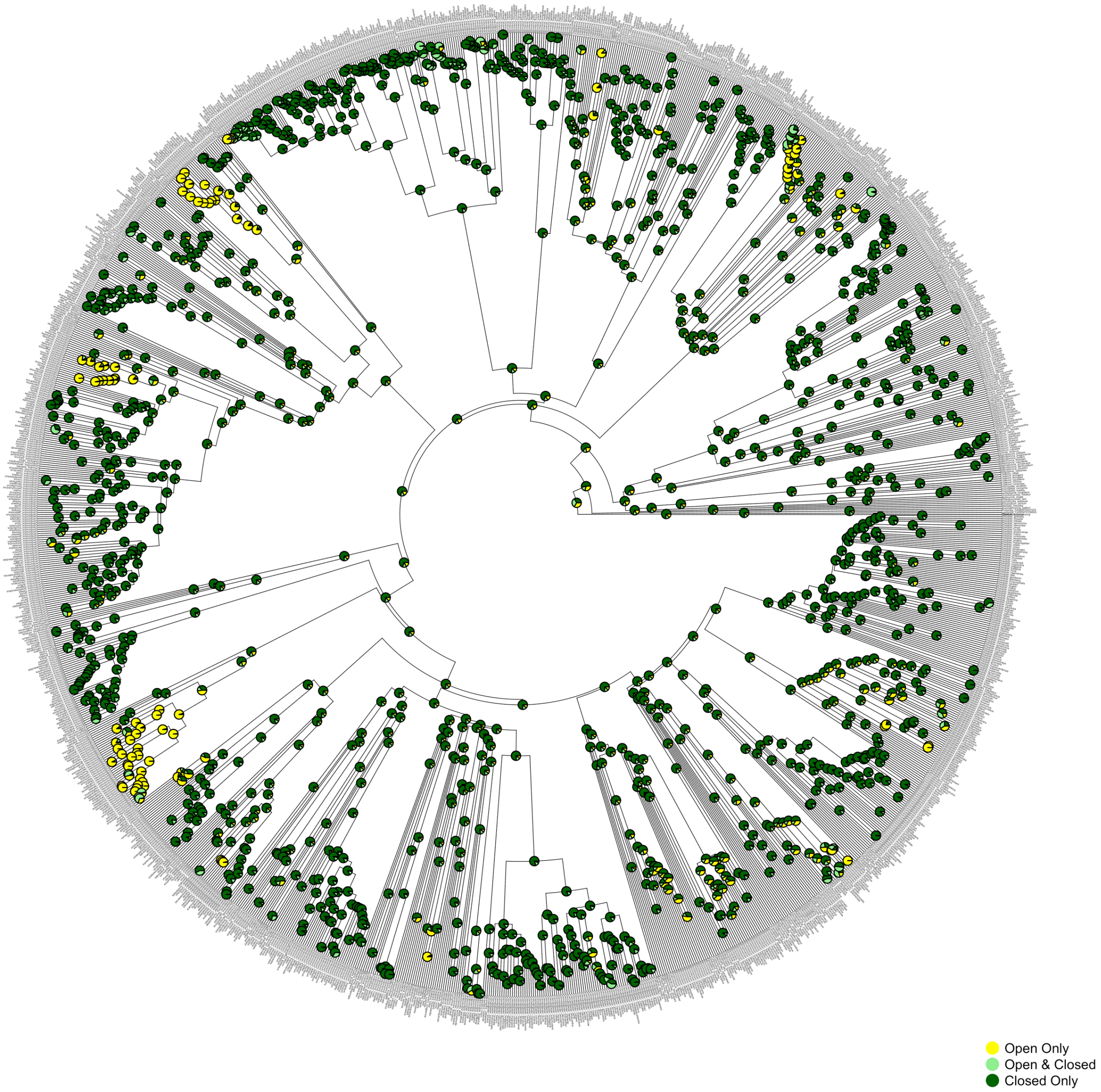

Figure S1. Time-scaled phylogeny of 1,435 ant species illustrating the evolution of ants in open- and closed-canopy habitats (non-forested and forested, respectively). Pie charts overlaying nodes indicate the proportional probability of occupying closed canopy, open canopy or mixed habitats. Node states are identical to those in Figure 1, but tip labels are provided here.

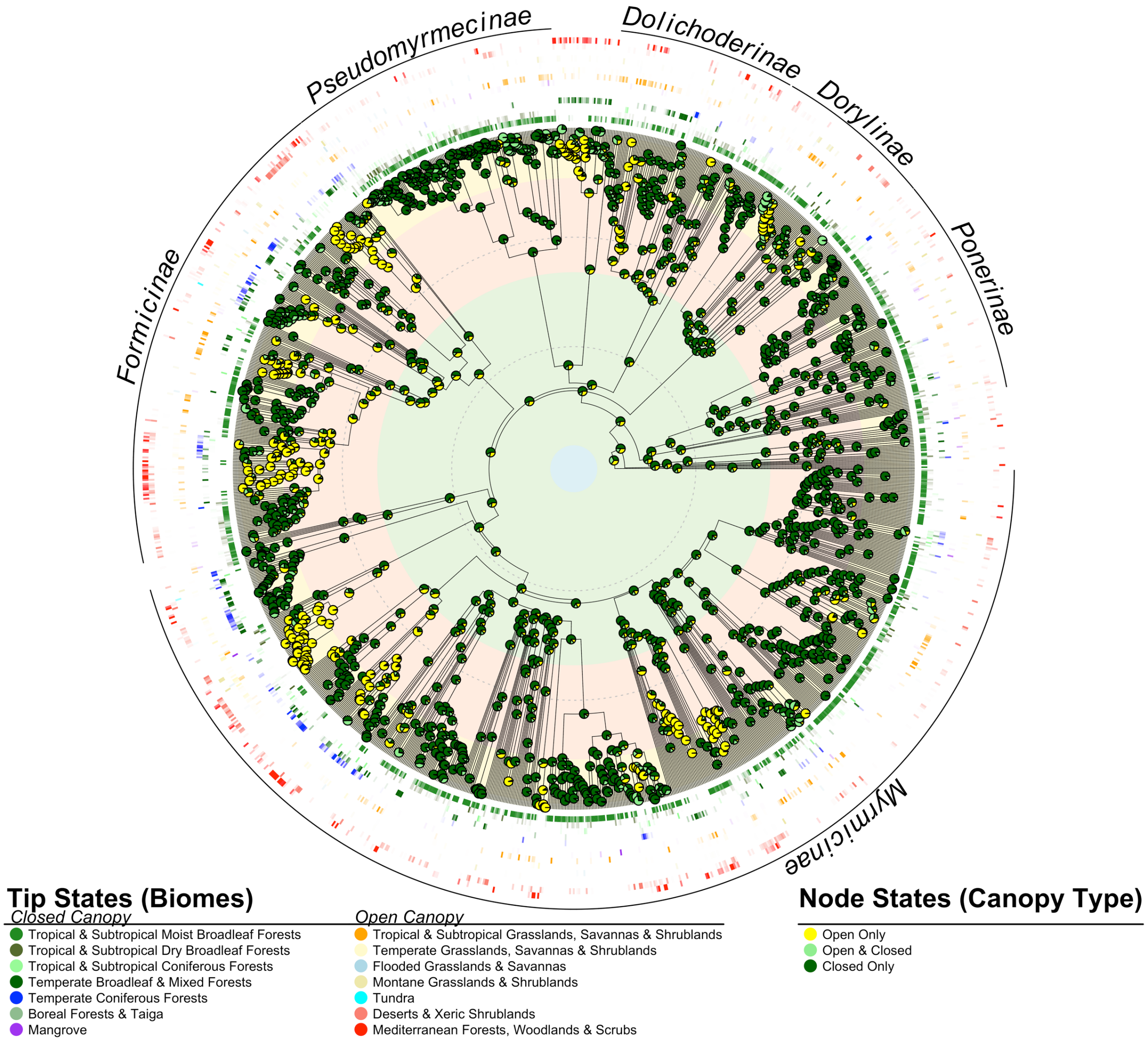

Figure S2. Time-scaled phylogeny of 1,435 ant species illustrating the evolution of ants in open- and closed-canopy habitats (non-forested and forested, respectively). In contrast to Figure 1 and Figure S2, Mediterranean Forests, Woodlands and Scrubs were treated as open-canopy habitats. Pie charts overlaying nodes indicate the proportional probability of occupying closed canopy, open canopy or mixed habitats. Rings around the tips of the phylogeny are colored by modern biome type and opaqueness reflects the proportion of specimens for individual species recovered from each biome. Shaded bands under the phylogeny correspond to geological periods, with dashed lines occurring in 50-Ma intervals.

Table S1. Environmental and soil variables and data layers used.

Abbreviation	Variable	Unit	Resolution	Years	Obtained from	Reference
BIO1	Annual Mean Temperature	°C	2.5 minutes	1960-1990	Worldclim v1.4 in dismo	Hijmans et al. (2005)
BIO2	Mean Diurnal Range (Mean of monthly (max temp - min temp))	°C	2.5 minutes	1960-1990	Worldclim v1.4 in dismo	Hijmans et al. (2005)
BIO3	Isothermality (BIO2/BIO7) (* 100)	°C	2.5 minutes	1960-1990	Worldclim v1.4 in dismo	Hijmans et al. (2005)
BIO4	Temperature Seasonality (standard deviation *100)	°C	2.5 minutes	1960-1990	Worldclim v1.4 in dismo	Hijmans et al. (2005)
BIO5	Max Temperature of Warmest Month	°C	2.5 minutes	1960-1990	Worldclim v1.4 in dismo	Hijmans et al. (2005)
BIO6	Min Temperature of Coldest Month	°C	2.5 minutes	1960-1990	Worldclim v1.4 in dismo	Hijmans et al. (2005)
BIO7	Temperature Annual Range (BIO5-BIO6)	°C	2.5 minutes	1960-1990	Worldclim v1.4 in dismo	Hijmans et al. (2005)
BIO8	Mean Temperature of Wettest Quarter	°C	2.5 minutes	1960-1990	Worldclim v1.4 in dismo	Hijmans et al. (2005)
BIO9	Mean Temperature of Driest Quarter	°C	2.5 minutes	1960-1990	Worldclim v1.4 in dismo	Hijmans et al. (2005)
BIO10	Mean Temperature of Warmest Quarter	°C	2.5 minutes	1960-1990	Worldclim v1.4 in dismo	Hijmans et al. (2005)
BIO11	Mean Temperature of Coldest Quarter	°C	2.5 minutes	1960-1990	Worldclim v1.4 in dismo	Hijmans et al. (2005)
BIO12	Annual Precipitation	mm	2.5 minutes	1960-1990	Worldclim v1.4 in dismo	Hijmans et al. (2005)
BIO13	Precipitation of Wettest Month	mm	2.5 minutes	1960-1990	Worldclim v1.4 in dismo	Hijmans et al. (2005)
BIO14	Precipitation of Driest Month	mm	2.5 minutes	1960-1990	Worldclim v1.4 in dismo	Hijmans et al. (2005)
BIO15	Precipitation Seasonality (Coefficient of Variation)	mm	2.5 minutes	1960-1990	Worldclim v1.4 in dismo	Hijmans et al. (2005)
BIO16	Precipitation of Wettest Quarter	mm	2.5 minutes	1960-1990	Worldclim v1.4 in dismo	Hijmans et al. (2005)
BIO17	Precipitation of Driest Quarter	mm	2.5 minutes	1960-1990	Worldclim v1.4 in dismo	Hijmans et al. (2005)
BIO18	Precipitation of Warmest Quarter	mm	2.5 minutes	1960-1990	Worldclim v1.4 in dismo	Hijmans et al. (2005)
BIO19	Precipitation of Coldest Quarter	mm	2.5 minutes	1960-1990	Worldclim v1.4 in dismo	Hijmans et al. (2005)
NPP	Mean Annual Net Primary Productivity	g C/m^2	30 seconds	2000-2015	Numerical Terradynamic Simulation Group (NTSG) synthesis of mean values from MOD17A3 v55, http://files.ntsg.umt.edu/data/NTSG_Products/MOD17/GeoTIFF/MOD17A3/GeoTIFF_30arcsec/	
PET	Annual Average Potential Evapo-Transpiration	mm	30 seconds	1950-2000	CGIAR-CSA Global Aridity Index and Global Potential Evapo-Transpiration Climate Database, https://cgiarcsi.community/data/global-aridity-and-pet-database/	Trabucco et al. (2009)
ELEV	Elevation	m	30 seconds		ArcGIS	
T_GRAVEL	Topsoil Gravel Content	% vol.	30 seconds		Harmonized World Soil Database v1.2 (2012)	Nachtergaele et al. (2012)
T_SAND	Topsoil Sand Fraction	% wt.	30 seconds		Harmonized World Soil Database v1.2 (2012)	Nachtergaele et al. (2012)
T_SILT	Topsoil Silt Fraction	% wt.	30 seconds		Harmonized World Soil Database v1.2 (2012)	Nachtergaele et al. (2012)
T_CLAY	Topsoil Clay Fraction	% wt.	30 seconds		Harmonized World Soil Database v1.2 (2012)	Nachtergaele et al. (2012)
T_BULK_DENSITY	Topsoil Reference Bulk Density	kg/dm^3	30 seconds		Harmonized World Soil Database v1.2 (2012)	Nachtergaele et al. (2012)
T_OC	Topsoil Organic Carbon	% weight	30 seconds		Harmonized World Soil Database v1.2 (2012)	Nachtergaele et al. (2012)
T_PH_H2O	Topsoil pH (H_20)	-log(H^+)	30 seconds		Harmonized World Soil Database v1.2 (2012)	Nachtergaele et al. (2012)
T_CEC_SOIL	Topsoil Cation Exchange Capacity (CEC) (Soil)	cmol/kg	30 seconds		Harmonized World Soil Database v1.2 (2012)	Nachtergaele et al. (2012)
T_BS	Topsoil Base Saturation	%	30 seconds		Harmonized World Soil Database v1.2 (2012)	Nachtergaele et al. (2012)
T_TEB	Topsoil Total Exchangeable Bases (TEB)	cmol/kg	30 seconds		Harmonized World Soil Database v1.2 (2012)	Nachtergaele et al. (2012)
T_CACO3	Topsoil Calcium Carbonate	% weight	30 seconds		Harmonized World Soil Database v1.2 (2012)	Nachtergaele et al. (2012)
T_CASO4	Topsoil Gypsum	% weight	30 seconds		Harmonized World Soil Database v1.2 (2012)	Nachtergaele et al. (2012)
T_ESP	Topsoil Exchangeable Sodium Percentage (ESP) (Sodicity)	%	30 seconds		Harmonized World Soil Database v1.2 (2012)	Nachtergaele et al. (2012)
T_ECE	Topsoil Electrical Conductivity (Elco) (Salinity)	dS/m	30 seconds		Harmonized World Soil Database v1.2 (2012)	Nachtergaele et al. (2012)

Table S2. Terrestrial biomes (Olson et al. 2001) classified by vegetation/canopy structure. Closed is regarded as forested habitat, while open is treated as non-forested habitat.

Vegetation Structure	Biome Type (Olson et al. 2001)	Shapefile Code
Closed	Tropical and subtropical moist broadleaf forests	A
Closed	Tropical and subtropical dry broadleaf forests	B
Closed	Tropical and subtropical coniferous forests	C
Closed	Temperate broadleaf and mixed forests	D
Closed	Temperate coniferous forests	E
Closed	Boreal forests and taiga	F
Closed	Mediterranean forests, woodlands and scrubs	L
Closed	Mangrove	N
Open	Tropical and subtropical grasslands, savannas and shrublands	G
Open	Temperate grasslands, savannas and shrublands	H
Open	Flooded grasslands and savannas	I
Open	Montane grasslands and shrublands	J
Open	Tundra	K
Open	Deserts and xeric shrublands	M

Table S3. Summary of the fits of models of evolution to different climatic variables. See Table S1 for explanation of variables. Measures include AICc values, difference in AICc values between best-fitting model and model evaluated (ΔAICc), and AICc weights ($AICc_w$).

Model	Measure	BIO1	BIO4	BIO6	BIO7	BIO11	BIO12
BM1	AICc	8752.40	19023.57	9731.71	9714.13	9476.77	23584.99
BM1	ΔAICc	623.65	373.31	406.45	436.50	432.80	536.20
BM1	$AICc_w$	0.00	0.00	0.00	0.00	0.00	0.00
BMS	AICc	8668.14	19025.29	9724.14	9714.88	9449.69	23560.72
BMS	ΔAICc	539.39	375.02	398.87	437.25	405.72	511.93
BMS	$AICc_w$	0.00	0.00	0.00	0.00	0.00	0.00
OU1	AICc	8226.10	18672.92	9356.71	9298.00	9097.71	23057.81
OU1	ΔAICc	97.34	22.66	31.45	20.37	53.74	9.02
OU1	$AICc_w$	0.00	0.00	0.00	0.00	0.00	0.01
OUM	AICc	8212.08	18667.51	9351.06	9294.19	9090.04	23051.76
OUM	ΔAICc	83.32	17.24	25.80	16.56	46.07	2.97
OUM	$AICc_w$	0.00	0.00	0.00	0.00	0.00	0.12
OUMV	AICc	8135.24	18660.13	9329.96	9284.03	9049.49	***23049.76***
OUMV	ΔAICc	6.48	9.87	4.70	6.40	5.52	***0.97***
OUMV	$AICc_w$	0.00	0.00	0.00	0.00	0.00	***0.33***
OUMA	AICc	8155.19	18666.47	9339.35	9290.67	9063.11	NA
OUMA	ΔAICc	26.44	16.20	14.09	13.04	19.15	NA
OUMA	$AICc_w$	0.04	0.01	0.09	0.04	0.06	NA
OUMVA	AICc	***8128.75***	***18650.26***	***9325.26***	***9277.63***	***9043.97***	***23048.79***
OUMVA	ΔAICc	***0.00***	***0.00***	***0.00***	***0.00***	***0.00***	***0.00***
OUMVA	$AICc_w$	***0.96***	***0.99***	***0.91***	***0.96***	***0.94***	***0.54***
