# Supplementary material for: Macroecological diversification of ants is linked to angiosperm evolution
Source: Evol Lett. 2023 Mar 31;7(2):79–87. doi: 10.1093/evlett/qrad008 (PMC10078972; doi:10.1093/evlett/qrad008)
Supplement: qrad008_suppl_Supplementary_Table_S4 [file qrad008_suppl_supplementary_table_s4.docx]

**Table S4.** Model fit metrics and parameter estimates for OUMVA models. Cells with three values represent the lower bound of the 95% CI (italics), the observed value (bold), and the upper bound of the 95% CI (italics). Variables include Annual Mean Temperature (BIO1), Temperature Seasonality (BIO4), Minimum Temperature of Coldest Month (BIO6), Mean Temperature of Coldest Quarter (BIO11) and Annual Precipitation (BIO12).

| Variable | lnL | AICc | AICc weight | θ_G_ | θ_A_ | α_G_ | α_A_ | σ^2^_G_ | σ^2^_A_ | Phylogenetic halflife_G_ | Phylogenetic halflife_A_ | Stationary variance_G_ | Stationary variance_A_ |
| --- | --- | --- | --- | --- | --- | --- | --- | --- | --- | --- | --- | --- | --- |
| BIO1 | -4058.35 | 8128.75 | 0.96 | *20.02-***20.32***-21.19* | *22.07-***22.35***-22.86* | *0.04-***0.04***-0.05* | *0.04-***0.05***-0.05* | *2.61-***2.82-**  *3.60* | *0.39-***0.70***-1.21* | *13.76-***17.29***-18.40* | *12.86-***15.15***-17.93* | *31.09-***35.19***-38.72* | *4.12-***7.68***-15.15* |
| BIO4 | -9319.10 | 18650.26 | 0.99 | *228.50-***267.20***-283.92* | *150.10-***185.19***-200.97* | *0.03-***0.03***-0.04* | *0.03-***0.04***-0.04* | *2913.36-***3168.87***-3974.12* | *506.41-***1116.09***-1465.50* | *17.34-***23.34***-24.87* | *17.15-***19.19***-24.54* | *44586.89-***1553350.55***-58582.83* | *7189.52-***15449.41***-25202.11* |
| BIO6 | -4656.60 | 9325.26 | 0.91 | *10.97-***11.44***-12.89* | *13.57-***14.25***-15.68* | *0.03-***0.03***-0.04* | *0.03-***0.03***-0.04* | *4.14-***4.72***-5.66* | *0.98-***1.92***-2.82* | *18.78-***22.64***-25.28* | *18.23-***19.83***-24.10* | *68.58-***77.12***-83.14* | *13.70-***27.46***-45.22* |
| BIO7 | -4632.79 | 9277.63 | 0.96 | *17.34-***18.49***-18.93* | *15.04-***16.27***-16.94* | *0.03-***0.03***-0.04* | *0.03-***0.04***-0.04* | *4.40-***4.68***-5.55* | *1.02-***1.94***-2.32* | *16.56-***19.96***-21.92* | *16.21-***17.47***-20.93* | *68.58-***67.43***-83.14* | *13.82-***24.43***-31.70* |
| BIO11 | -4515.95 | 9043.97 | 0.94 | *16.42-***17.01***-18.47* | *19.41-***19.93***-21.29* | *0.03-***0.03***-0.04* | *0.03-***0.04***-0.04* | *3.78-***4.17***-5.07* | *0.66-***1.37***-1.76* | *17.70-***22.42***-24.18* | *17.71-***19.31***-22.81* | *60.34-***67.44***-74.69* | *9.37-***19.08***-24.87* |
| BIO12 | -11518.37 | 23048.79 | 0.54 | *1585.68-***1657.25***-1762.16* | *1774.56-***1907.82***-2066.55* | *0.04-***0.05***-0.06* | *0.04-***0.05***-0.06* | *67233.85-***80140.29***-94345.78* | *84197.23-***137092.44***-2111934.05* | *11.55-***13.94***-16.55* | *11.37-***14.87***-16.40* | *717873.90-***806063.52***-902351.70* | *800124.70-***1470601.40***-2487635.30* |
